# “Okay, you’re moving to the adult world”: perspectives on transitioning users of home mechanical ventilation to adult care

**DOI:** 10.1093/annalsats/aaoag108

**Published:** 2026-04-27

**Authors:** Jessica M R Friesen, Kerry Koke, Marie F A Wright

**Affiliations:** Division of Paediatric Respiratory Medicine, BC Children’s Hospital, Vancouver, BC, Canada; Pediatric Respiratory Research Team, BC Children’s Hospital Research Institute, Vancouver, BC, Canada; Division of Paediatric Respiratory Medicine, BC Children’s Hospital, Vancouver, BC, Canada; Division of Paediatric Respiratory Medicine, BC Children’s Hospital, Vancouver, BC, Canada; Pediatric Respiratory Research Team, BC Children’s Hospital Research Institute, Vancouver, BC, Canada; Department of Pediatrics, University of British Columbia, Vancouver, BC, Canada

**Keywords:** home mechanical ventilation, pediatric to adult transition, qualitative research

## Abstract

**Rationale:**

Children requiring long-term home mechanical ventilation (HMV) increasingly survive into adulthood. However, structured processes to support their transition from pediatric to adult care are lacking. Understanding the perspectives of patients, caregivers, and health and social care providers (HSCPs) is critical to improving transition experiences and outcomes.

**Objectives:**

To explore the experiences, perceptions, and priorities of patients, caregivers, and providers involved in the transition of young people using HMV from pediatric to adult care, and to identify practical targets for improvement.

**Methods:**

We conducted semi-structured interviews and focus groups with the following participant groups: pediatric and adult HSCPs (*n* = 14) and patients aged 17 to 21 years with experience of HMV (*n* = 7) and their caregivers (*n* = 9). Iterative inductive thematic analysis was performed. Patient partners with lived experience contributed to study design, development of data collection tools, and analysis.

**Results:**

Four key themes emerged: (1) The absence of a formal transition framework left patients and caregivers unclear about expectations, timelines, and responsibilities; (2) care continuity and coordination were often disrupted, with prolonged gaps between pediatric and adult care and confusion over roles of providers; (3) patients were insufficiently prepared for differences in adult care, including greater self-management expectations; and (4) providers reported systemic barriers, including lack of institutional support, insufficient resources, and overreliance on individual “transition champions” to drive processes. These challenges were consistently reported across participant groups.

**Conclusions:**

Young people using HMV face significant challenges during and after their transition to adult care. Priorities for improvement include the need for structured transition protocols, early planning, education about adult care expectations, and formalized handovers between services. Sustainable improvements require operational support and integration of transition planning into routine care from early adolescence. Future research should evaluate the impact of formalized transition models on patient experiences and outcomes.

## Introduction

Children who use long-term home mechanical ventilation (HMV) are a medically complex and diverse population including individuals with neuromuscular disorders, multitudinous genetic syndromes, and primary disorders of the respiratory system. Pediatric users of HMV may use noninvasive respiratory support—continuous positive airway pressure (CPAP) or bilevel positive airway pressure (BPAP)—or invasive ventilation via tracheostomy depending on factors including their underlying medical condition, level of ventilation dependency, and patient/caregiver preferences. Pediatric HMV use is expanding globally due to advancements in ventilator technology, emphasis on supporting children at home rather than inpatient healthcare settings, and improved survival of children with medical complexity. The rapidly growing size and increasing life expectancy of this population has introduced novel challenges, including the need to navigate transition from pediatric to adult medical and social care systems.

Transition to adult medical services is defined as the “purposeful, planned movement of adolescents with chronic medical conditions from child-centred to adult-oriented health care.”^1^ This process of transition is a necessary step in ensuring that young adults receive care that meets their changing needs, as pediatric centers may lack the expertise and resources to support areas such as reproductive health, autonomy, and self-advocacy, and may not be adequately trained to manage the medical problems encountered by adult patients.^2^^,^^3^ As survival of pediatric users of HMV into adulthood has become increasingly common, transition planning and establishment of equivalent adult medical services have failed to keep up with demand for this rapidly growing medical field.^4-6^ Adverse outcomes including poor adherence to treatments and increased illness severity, hospitalization rates, and mortality have been described in young adults with medical complexity around the time of transfer of care to adult services, illustrating the importance of providing a high standard of care during this formative period.^6-9^ Transition can also place a significant burden on healthcare resources, as illustrated by a doubling in hospital admissions via the emergency department around the time of care transfer to adult services in young adults with congenital heart disease.^10^ Thus, improving transition processes may address challenges at both the individual and systemic levels.

Literature outlining the process and impact of transition among young adult users of HMV is limited, and even fewer studies describe patient, caregiver, and provider perspectives on transition. By identifying common themes within the experiences of patients, caregivers, and health and social care providers (HSCPs) of transitioning from pediatric to adult HMV care, our study aimed to improve current understanding of how transition impacts these key individuals and to use these insights to make practical recommendations about how to improve this process.

## Methods

### Study design

We conducted a qualitative research study using an interpretative description methodology, which applies inductive analysis to generate practice-relevant insights that can inform clinical care and policy.^11^ This study was carried out as part of a larger quality improvement (QI) initiative to improve the delivery of transition care within a single pediatric HMV program. The qualitative data collected formed a key part of the current state analysis and future state planning components of the project.

### Setting

The study was conducted within the pediatric HMV program at BC Children’s Hospital (BCCH), Vancouver, Canada, which supports children and youth across British Columbia (BC) requiring home invasive or noninvasive positive airway pressure therapy for conditions such as parenchymal lung disease, neuromuscular weakness, airway obstruction, and respiratory drive disorders. At our institution, approximately three-quarters of pediatric HMV patients use noninvasive forms of respiratory support (CPAP or BPAP) rather than tracheostomy ventilation, and this pattern was reflected in the composition of our study sample. Patients typically transfer to adult care by high school graduation, most often to an adult respirologist, sleep physician, or primary care provider. Those using invasive ventilation or noninvasive BPAP receive community respiratory therapy support, whereas CPAP users do not. At the time of the study, no formal transition framework existed; transition planning was directed individually by the pediatric respirologist at each clinic visit. Specialist care was typically sequential rather than collaborative, with patients graduating from the pediatric clinic prior to their first consultation in the adult medical clinic, without an established process for joint or overlapping clinic visits. The use of formal transition readiness assessment tools was not a standard practice and would be used at individual provider discretion.

### Study team

Our team included a pediatric respirologist and nurse practitioner with expertise in pediatric HMV and a background of formal QI training, a QI leader and administrator, and 2 patient partners with lived experience of HMV use and transition to adult care. Patient partners were involved in study design, interview script development, and data analysis.

### Study participants

We conducted semi-structured interviews and focus groups with (1) patients/caregivers of the BCCH HMV clinic who used either invasive or noninvasive forms of HMV and (2) HSCPs working in hospital inpatient, outpatient, and community settings within BC.

Eligible patient/caregiver participants were current patients within 1 year of anticipated transfer to adult services (age 17-18 years) or those who had transferred within the past 3 years (age 18-21 years), along with their primary caregivers. When patients were unable to participate due to severe intellectual disability or major communication barriers, caregivers were invited to participate independently.

HSCP participants were purposively sampled based on their clinical expertise in supporting pediatric HMV users, or patients with other complex and chronic medical conditions, through transition from pediatric to adult care. Many participants served as transition leads or informal transition “champions” within their clinical areas. Community-based primary care providers were also invited to participate, recognizing their important role in transition care; however, despite recruitment efforts through multiple channels, no primary care providers were enrolled within the study timeframe.

A summary of study participants and interview types is provided in Figure 1, and HSCP backgrounds are shown in Table S1.

**Figure 1:**
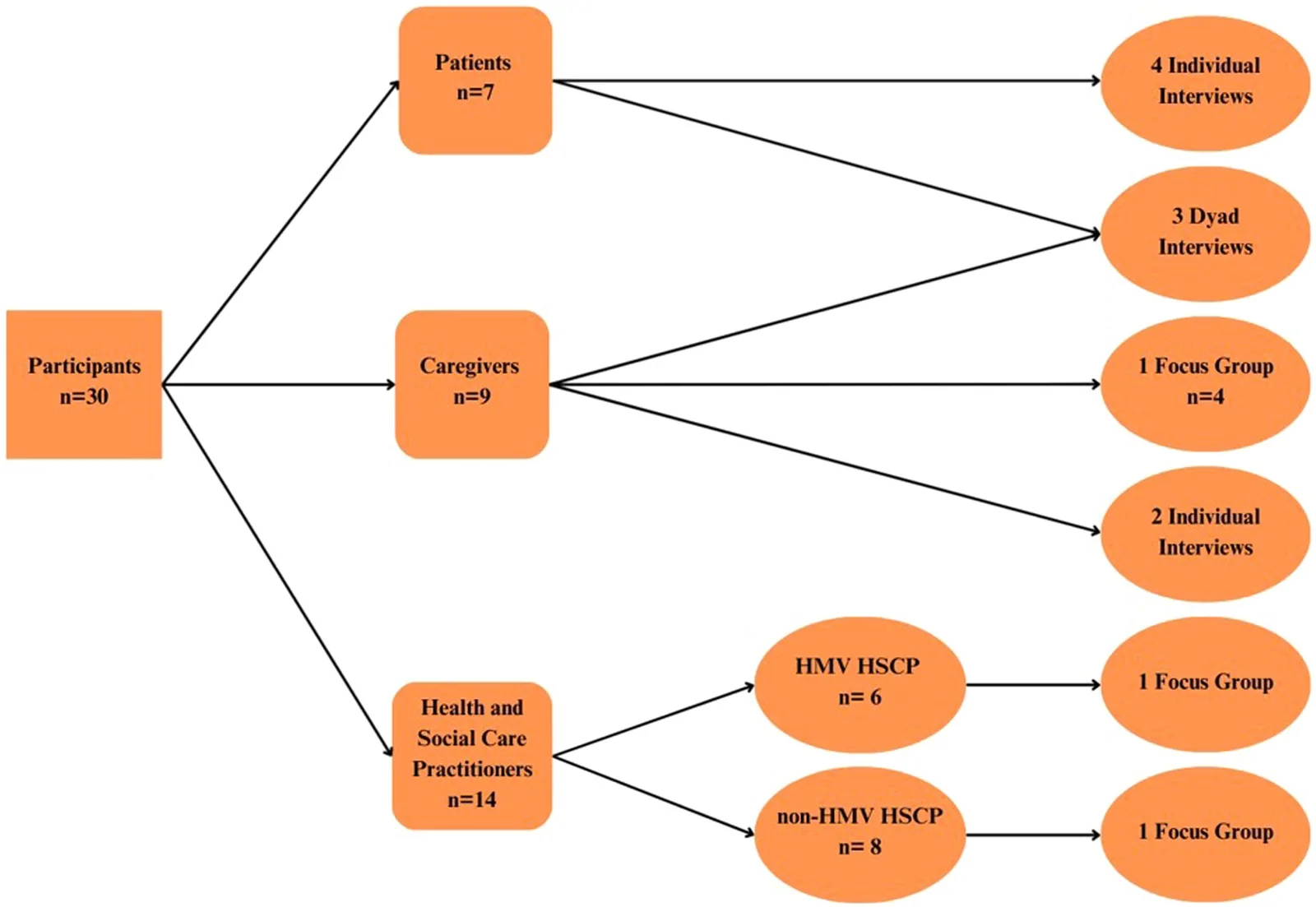
Flowchart of interview participants and formats. Abbreviations: HMV, home mechanical ventilation; HSCP, health and social care provider.

Convenience sampling was used. Patient and caregiver participants were identified through the pediatric HMV clinic database and contacted initially by email, with follow-up by telephone for nonresponders. Translator-supported telephone or video interviews were offered to non-English speakers. Participation was voluntary, and all individuals who agreed were included. HSCPs were approached by the study team to ensure representation of key provider groups, with invitations issued by email or direct conversation.

All participants provided consent for their interview to be recorded prior to their interview. Patient/caregiver participants received a $50 gift card after completion of the interview.

### Data collection and analysis

#### Interviews

A semi-structured interview guide was developed by the study team, informed by current literature on transitions in children with medical complexity and a chart review of recent pediatric-to-adult transitions within the BCCH HMV clinic. The literature review was conducted using the University of British Columbia library databases that include articles from all major health and science journals including PubMed, Embase (Ovid), and Scopus. Search terms included “transition*,” “home mechanical ventilation,” “neuromuscular,” and “sleep medicine.” Topics in the interview guide included perceptions of transition success; experiences of successful and challenging transitions; interprovider and patient–provider communication; and the key providers, education, and resources required to support the transition process. We piloted the interview guide with patient partners, and these pilot interviews were included in the qualitative analysis.

Interviews were conducted between February and April 2023, lasting 30 to 60 minutes for individual interviews and up to 2 hours for focus groups. All sessions were conducted virtually via Zoom for Healthcare (hosted by the Provincial Health Services Authority of BC) by an external facilitator and researcher with no clinical relationship to participants. Transcripts were generated using automated transcription software.

#### Data analysis

Thematic analysis was conducted using an iterative, inductive approach. Transcripts were manually edited, de-identified, and reviewed by 2 study team members for familiarity. Coding was performed in NVivo 12 (Lumivero, Denver, CO, USA), with initial codes generated directly from transcript content and refined into overarching themes through repeated review cycles and team discussion.

## Results

Thirty individuals were interviewed across 4 participant categories: HMV-focused providers (HMV HSCPs, *n* = 6), non-HMV providers (non-HMV HSCPs, *n* = 8), patients (*n* = 7), and caregivers (*n* = 9). All invited HSCPs participated or identified a delegate from their team. Of 24 eligible patient/caregiver dyads, 13 (54%) dyads were represented in the interviews. Three participated as a dyad (*n* = 6 participants) and in 10 instances either the participant (*n* = 4) or the caregiver (*n* = 6) participated independently (Figure 1).

Within the patient/caregiver cohort, the most common primary diagnosis was a neuromuscular disorder (38%, *n* = 5). Four participants had a known genetic syndrome (Trisomy 21, Marshall–Smith, Marfan, Joubert). Eighty-five percent (*n* = 11) were noninvasively ventilated and 2 were ventilated via a tracheostomy. Fifty-four percent (*n* = 7) had already graduated from the pediatric clinic and 46% (*n* = 6) were in the process of transitioning to adult services.

Participant responses to interview questions were categorized using parent (*n* = 8) and child (sub)codes (*n* = 18) (Table S2). Four key themes were identified (Table 1). The most common words spoken by interview participants are illustrated in Figure 2.

**Figure 2:**
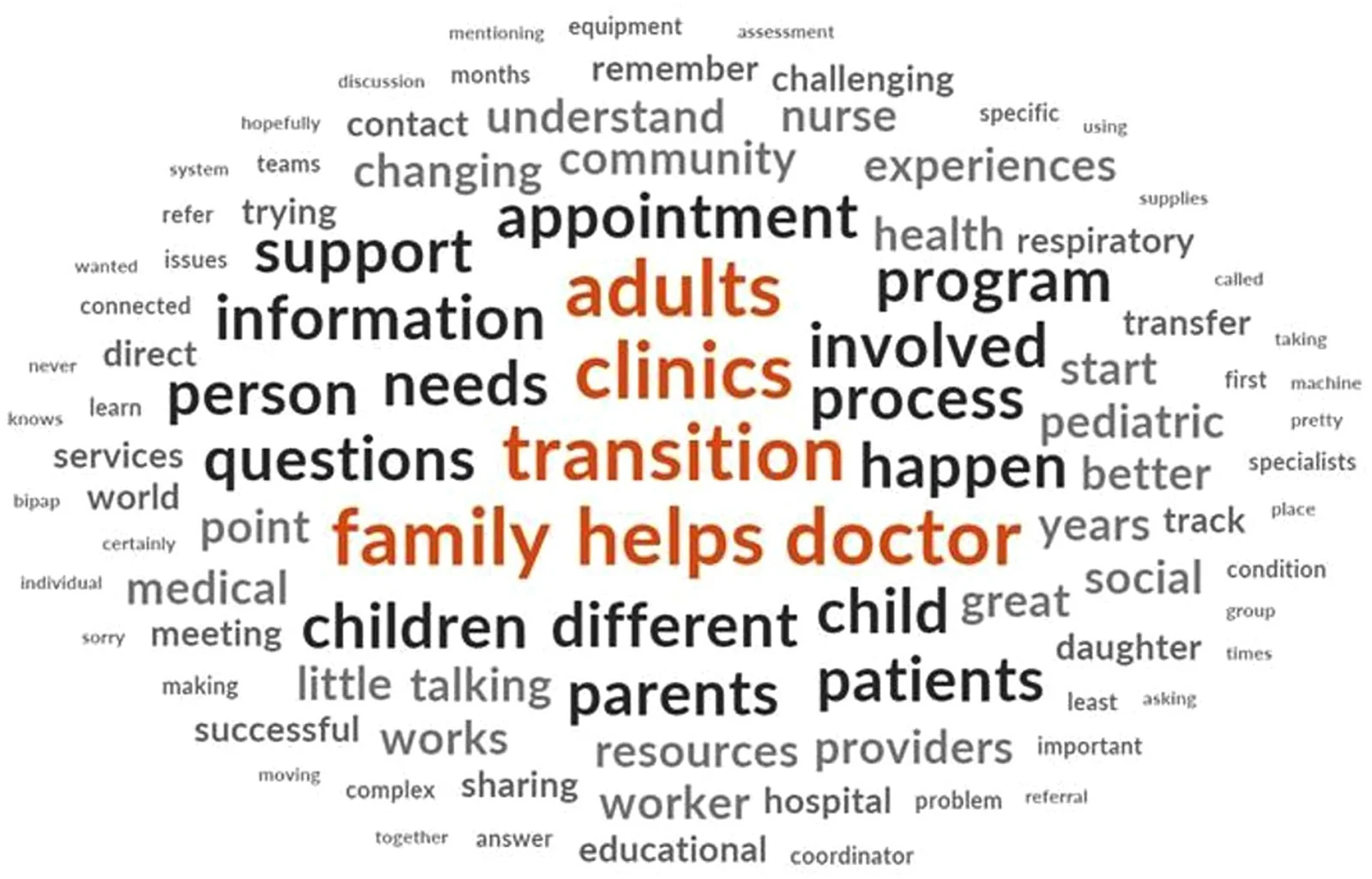
Word cloud depicting the 100 most common words spoken by participants during interviews.

**Table 1:** Key themes from interviews and focus group discussion.

**All participant groups**	Theme 1	When transition lacks formal structure, patients/caregivers do not have a clear understanding or expectation of the process.
	Theme 2	A lack of care continuity and coordination is experienced by patients/caregivers and providers during transition.
	Theme 3	Readiness for transition and expectations around differences within the adult medical system are not adequately addressed by providers.
**HSCP**	Theme 4	Healthcare providers are not able to address all needs of the population during transition due to systemic barriers.

Abbreviation: HSCP, health and social care provider.

### Theme 1: When transition lacks formal structure, patients/caregivers do not have a clear understanding or expectation of the process

*“The communication has been more reactive in the sense of ‘Oh, do you have an OT?’ ‘No, I don’t think so.’ ‘Oh, let me make a referral for you, you should have that.’ … and then it’s like, were they supposed to call us? We’re like, who were they again? I don’t remember. How do we contact them?”* —Participant 22, Caregiver

Participants across all groups described their experience of transition as being “inefficient,” “unclear,” and “not smooth.” Patients and caregivers expressed that the communication and planning delivered by their care team was reactive rather than structured and preemptive. Providers agreed that discussions around transition were often on an ad hoc basis and did not follow a clear structure or timeline (Table 2A, Quote 1). Patients/caregivers were consequently left to navigate transition without understanding what they needed to accomplish before transfer to adult care, and without resources needed to support their child through the process (Table 2A, Quote 2).

**Table 2:** Illustrative interview quotes.

		Quote	Group	*n*
**(A) Theme 1**	1	Often, it’s like the last appointment. We’re like, “okay, you’re moving to the adult world, here’s your referral for your respirologist” and … we see [the patient] a year later like, “yeah, we still haven’t seen [the adult respirologist].”	HMV HSCP	6
	2	I think there are organizations that help people with [disabilities]. But if you don’t know about them in advance, then you wouldn’t even know to look for them.	Caregiver	16
	3	Like, a family resource tool that clearly explains eligibility and […] where to find who does what.	HMV HSCP	3
**(B) Theme 2**	1	Recently it’s been quite frustrating because I’ve been dealing with … symptoms that could be related to [one of my diagnoses]. I’m not sure. But it’s so difficult to get in with [my family doctor] at all. So yeah, I don’t have any specialist care or barely any care from my family doctor.	Patient	2
	2	Probably [would have been good to have some overlap] just so that [my family doctor] at least has some information, like she really doesn’t have any information on my sleep apnea diagnosis. Honestly, I don’t think we’ve ever really talked about it.	Patient	2
	3	No one’s really taking an assessment of how realistic [it is] to dump everything on a [family doctor] in the community when they transition out of [the children’s hospital].	Caregiver	16
**(C) Theme 3**	1	I know in the pediatric care, […] you do feel extremely supported, and there is, you know, an element of handholding and collaboration, and you do feel like you’re part of a community and team. And now it feels like we are on our own and scrambling to figure out.	Caregiver	21
	2	When I turned 18, I had to make all the decisions and all these calls and all these things … I’m making so many decisions that I’ve never made.	Patient	1
	3	Like my breathing changed. That part I did not know.	Patient	28
	4	The challenge is that a lot of [transitioning HMV users] are not going to […] meet the developmental milestones of becoming independent. So, it’s also trying to help parents sort out what it’s going to look like as they get older and less physically able to care for their dependent children.	Non-HMV HSCP	9
	5	We don’t really have the luxury sometimes of being able to truly accept that a patient may or may not be ready when there’s this hard and fast cut-off that exists around how the system will change whether you like it or not.	Non-HMV HSCP	12
	6	If you look at things like high school or university, there’s a little bit of play in terms of when someone may graduate. But for the vast majority, people are graduating at age 17, 18, maybe 19, and it’s my personal belief that that should be the same for our transition programs. But with the huge proviso that, [in] the adult system, there have to be transition supports there also.	Non-HMV HSCP	10
	7	These specialists are all different places, and it’s really hard for them. Kids are getting older. They’re requiring more care. And now they’re going to all over the different hospitals.	Non-HMV HSCP	9
	8	All the specialties are very siloed, and it’s maybe like an orchestra. You need a conductor, kinda like our general pediatrician, or somebody who would just pull everything together. So it can just flow better. And somebody equivalent in adults.	Caregiver	17

Abbreviations: HMV, home mechanical ventilation; HSCP, health and social care provider.

Participants agreed that resources such as transition guides and timelines would make the process more transparent and easier to navigate, though preferences for paper versus digital formats varied. HSCP additionally emphasized the need for resources addressing differences in eligibility for funding and community-based services (Table 2A, Quote 3).

*“We need somewhere where people can go and say, ‘okay, what is available to me in the adult world? How do I apply for product distribution for these supplies that I can’t get covered anymore?’ Like some centralized […] location for information that people can go to would be a great resource. I mean for us [allied health professionals] and for physicians … families … because honestly sometimes … we don’t know what resources are out there for them.”* —Participant 7, HMV HSCP

### Theme 2: A lack of care continuity and coordination is experienced by patients/caregivers and providers during transition

*“Not having support for a year is difficult, especially if you need certain things—you should get [them] while you still have support.”* —Participant 1, Patient

A lack of care continuity between pediatric and adult services was commonly reported by patients/caregivers. Most reported a 6- to 12-month interval between discharge from the pediatric clinic and the first adult clinic appointment, during which they were unsure how to access medical services, how to obtain supplies, or where to go in an emergency. Participants recognized the potential detrimental impact of these care gaps on their health (Table 2B, Quote 1).

When first reviewed in the adult clinic, patients/caregivers perceived that the provider was inadequately informed about their medical needs, further delaying access to the support they needed. Patients/caregivers expressed that overlap between adult and pediatric clinic visits before transfer of care would improve information-sharing between professionals and reduce care gaps (Table 2B, Quote 2).

Patients graduating from specialized pediatric clinics are not always transferred to an adult specialist and in some cases are followed only by their family doctor after pediatric clinic graduation. Some patients/caregivers expressed that the family doctor had minimal involvement in their respiratory care prior to their graduation from the pediatric clinic, and questioned whether they had the resources and expertise to manage their complex medical needs (Table 2B, Quote 3). Caregivers identified the need for cross-clinic transition planning and coordination for children with complex chronic medical conditions:

*“There’s no clinic that totally coordinates the entire child. That looks at the big picture and tries to coordinate that. So, I know this goes beyond HMV, but they need to create a transition program for kids. That actually does this planning.”* —Participant 16, Caregiver

### Theme 3: Readiness for transition and expectations around differences within the adult medical system are not adequately addressed by providers

*“Our experience is that everybody says the transition is coming, but that’s all they say.”* —Participant 15, Caregiver

Participants from all groups agreed that a key factor in successful transfer of care is ensuring the patient and their caregivers are prepared, and that this is often inadequately addressed within the pediatric clinic. Parents/caregivers specifically voiced feeling unprepared for the differences in support and care coordination provided by pediatric and adult clinics (Table 2C, Quote 1) and the shift from medical decision-making and patient advocacy being caregiver-driven to being the responsibility of the patient (Table 2C, Quote 2). Further, patients expressed that they had not been adequately educated by their pediatric providers about the trajectory of their medical condition and how their health would change over time (Table 2C, Quote 3). Providers also emphasized that the health and aging of caregivers is an important consideration in transition planning, yet it is rarely discussed in pediatric clinics (Table 2C, Quote 4).

Although providers were unanimous that establishing patient readiness is central to a successful transition process, opinions regarding timing of care transfer varied. Some providers prioritized cognitive ability and individualized assessments of patient readiness over chronological age, expressing frustration that age-based cutoffs often dictate the timing of care transfer in practice, while others emphasized the importance of transferring by 18 years to support social development. Overall, providers highlighted the absence of clear guidance on how to balance individualized readiness with system-driven age-based expectations (Table 2C, Quotes 5 and 6).

Participants from all groups recognized that care is more fragmented within the adult system, resulting in additional workload for patients and caregivers who assume responsibility for care coordination and communicating plans between providers (Table 2C, Quotes 7 and 8). Given that differences in healthcare models between pediatric and adult clinics are inevitable, preparing the patient for these changes in advance of their transition is paramount:

*“… that understanding of responsibility, of taking ownership for some of their care needs is […] a difficult transition, right? People get into the habit of being contacted when they’re due for their six-month dental planning. But [adult medical providers] don’t operate that way, and I think that’s probably the one of the biggest things for them is to understand that we don’t operate that way.”* —Participant 7, HMV HSCP

### Theme 4: Healthcare providers are not able to address all needs of the population during transition due to systemic barriers

*“Most transition protocols are completely driven by one health care professional, and if that health care professional leaves or retires […] the whole thing could fall apart.”* —Participant 10, non-HMV HSCP

HSCPs identified many gaps in their capacity to facilitate a successful transition for their patients. Many practitioners believed that they lacked the time or resources to be able to provide a smooth transition experience in their clinic. They expressed that transition is commonly taken on by one person in a clinic rather than being a shared responsibility and embedded in the standard operating procedure of the clinic.

Providers noted that the transition lead role within their teams was typically assumed informally, without protected time or recognition in workforce planning. They emphasized that transition support was neither adequately staffed nor systematically integrated into service delivery. Across both pediatric and adult programs, HSCPs expressed concern that hospital leadership often overlooked the development and implementation of formal transition frameworks, despite the complex needs of this growing patient population. There was consensus that the demands associated with transitioning patients using HMV cannot be managed by caregivers alone:

*“There has to be institutional support for transition.”* —Participant 10, non-HMV HSCP

## Discussion

This study explores the experiences and perceptions of patients, caregivers, and HSCPs of transitioning from pediatric to adult HMV care. Across all groups, participants described major gaps and challenges in the transition process. Key issues included the absence of a structured framework (Theme 1), poor care continuity and support for families (Theme 2), inadequate preparation for the adult care model (Theme 3), and system-level barriers and oversights that leave providers underequipped to support patients (Theme 4). Collectively, these gaps contribute to patient vulnerability and highlight the urgent need for sustainable, structured transition pathways and operational support for transition within healthcare systems.

Our finding that transition to adult care is inadequately addressed for adolescents using HMV aligns with previous research.^6^^,^^12-16^ Dale and colleagues, in a study from another Canadian province, identified key barriers including poor coordination between pediatric and adult services, limited focus on assessing patient readiness, and insufficient guidance regarding community health resources and adult funding models.^6^ These same challenges were echoed in our study by both patients/caregivers and providers.

Setting expectations around adult care delivery, including the greater emphasis on patient-directed care and patient involvement in decision-making that is typical of adult healthcare systems, is a key principle of successful transition.^4^^,^^5^^,^^13^ Within our population, these expectations were not sufficiently communicated, highlighting the need for structured transition frameworks that include education about adult healthcare systems and support the development of self-advocacy skills. Ideally, these frameworks and adult care models should be flexible and adaptable, recognizing that some individuals with progressive conditions or significant functional or cognitive impairment will continue to require caregiver involvement in decision-making after transfer to adult care.

Adolescents transferring to adult care are known to face heightened risk of health decline, and provision of care continuity during and beyond transition can mitigate this risk.^4^^,^^7^^,^^12^^,^^13^^,^^17^ Our study confirms that care continuity is negatively impacted during transition to adult services for HMV users. To address this, transition frameworks should actively promote communication across pediatric and adult teams, and between primary care and specialist services. Potential strategies to achieve this include joint transition clinics, comprehensive handover documentation, and periods of overlapping care. Informed by the findings of this study, we have implemented a series of practice changes at our institution to strengthen transition care for pediatric HMV users. These initiatives, which may be adapted by other pediatric HMV programs, focus on improving consistency in transition processes, preparing patients for changes in their care model, strengthening communication across pediatric and adult providers, and ensuring access to practical resources to support both patients and clinicians throughout the transition period (Box 1).

Box 1 Practical strategies to improve transition care for pediatric home mechanical ventilation (HMV) users.

**Patient readiness and skill development** 

- Introduce progressive independence in clinic visits from early adolescence (eg, 14 years), with increasing patient-led participation where possible.
- Use HMV-specific transition readiness questionnaires at key ages (eg, 15 and 17 years) to guide individualized planning and education.
- Provide age-appropriate education on medical conditions, respiratory equipment, and daily care tasks, revisited over time.

**Communication and continuity of care** 

- Arrange overlapping pediatric and adult respirology visits during transition to support continuity and shared decision-making.
- Provide a patient “graduation pack” at transfer, documenting a concise medical summary, key investigations, current equipment and medications, and adult provider contact details.

**Patient and caregiver support** 

- Offer a dedicated transition planning visit with nursing and social work to address psychosocial needs and care coordination.
- Provide a written transition guide outlining timelines, expectations, and relevant community and adult-care resources.

**Provider and program support** 

- Implement a standardized transition framework with age-based task checklists to promote consistency across providers.
- Maintain a directory of adult care providers to support timely referrals.
- Embed transition documentation and referral templates in the electronic medical record.
- Hold periodic multidisciplinary transition review meetings (eg, every 6 months) to track individual patient progress and iteratively refine the transition framework.

While some of the areas for improvement identified in this study may be addressed at the level of individual HMV teams, addressing system-level issues requires engagement and investment from healthcare leadership. A scarcity of adult HMV providers means that some graduates from pediatric HMV programs are only followed by a primary care provider without appropriate resources or training to address their medical needs, and without the support of an allied health team.^13-15^ This situation will likely become more common as children with medical complexity are increasingly surviving into adulthood and being supported with HMV, and raises the question of whether increased investment in multidisciplinary clinics and complex care programs for adult patients is needed to effectively support this growing patient population.

The themes and change priorities identified by this study echo recommendations from current guidelines on transition of youth with complex healthcare needs.^3^^,^^5^^,^^18^ The Canadian Pediatric Society’s recommendations for transition of children with medical complexity include early and individualized transition planning using a stepwise and systematic approach that builds patient autonomy and provides care continuity during the care transfer process. The importance of systemic support for transition, for example through adaptable program funding and modified physician compensation systems to encourage integrated care, was also highlighted.^18^ The Canadian Thoracic Society (CTS) clinical practice guideline on pediatric HMV similarly recommends applying a structured transition process that starts around age 14, builds independence within the limits of the patient’s medical condition, and is supported collaboratively by pediatric and adult medical teams.^3^ In alignment with our findings, the CTS guideline identified that education and tools must be directed toward HSCPs, patients, and caregivers to facilitate a cohesive and coordinated transition.

In our study, HSCP participants reported similar challenges around patient transition irrespective of whether their background was in HMV or a non-HMV specialty. This is aligned with published studies that broadly identify similar key challenges and opportunities for improvement in transition across various patient groups and disease types. For example, communication, planning, and patient support have been identified as areas for improvement within transition programs for young people with cystic fibrosis, diabetes, and physical disabilities.^7^^,^^9^^,^^19^ The fact that transition care remains imperfect within patient groups that have published transition guidelines, dedicated patient and provider-facing transition resources, and established equivalent adult services highlights the scale of the task to optimize transition processes within the newer and evolving field of HMV.

This study has several strengths. Involvement of patient partners enhances the relevance and validity of research.^20^^,^^21^ We employed 2 patient partners with lived experience of HMV use and transfer of care to adult services who contributed to the study design, interview guide development, and data analysis. Their involvement ensured that each aspect of this study was patient-centered and addressed issues of greatest importance to our patient population and their caregivers.

Unlike previous studies focused solely on patient and caregiver experiences,^6^^,^^14^ our study also captured the perspectives of providers. This broader approach identified not only the challenges and priorities voiced by families but also the system-level barriers faced by providers about which patients/caregivers may be unaware. Strikingly, despite approaching transition from different viewpoints, both groups identified similar challenges and shared priorities for improvement.

Provider participants came from various fields of health and social care that support patients with medical complexity and technology dependency. This enabled us to look holistically at transition rather than focusing solely on experiences within the HMV clinic setting. It also enabled shared reflection and learning between HSCPs involved in different aspects of patient care who may not fully recognize the challenges encountered by other providers.

Rather than relying solely on a dyad approach, we also conducted individual interviews with the person responsible for directing care in the adult system. This allowed inclusion of caregivers of young adults unable to direct their own care, such as those with significant intellectual disability, recognizing that transition can be especially complex for this patient group. Given the diversity of this population in terms of medical diagnoses, physical function, and cognitive ability, a rigid interview format might have limited the participation of certain subgroups and introduced selection bias.

This study has limitations, including a small sample size and a heterogeneous patient population, which may reduce generalizability. However, because medical diversity is characteristic of pediatric HMV programs,^22^^,^^23^ our sample likely reflects the broader adolescent HMV population. Our study sample predominantly represented patients who use noninvasive HMV, which is consistent with the approach to pediatric HMV in our population. However, this may limit the generalizability to other populations where tracheostomy ventilation is favored over noninvasive therapies. It may also potentially minimize the challenges experienced by caregivers of HMV users, given that the burden of care associated with tracheostomy ventilation is reported as greater than with noninvasive ventilation,^24^ and may fail to identify some challenges that are unique to transitioning young adults who have tracheostomies. Recruitment of large cohorts remains challenging in this relatively small population, particularly within single-center studies. To enhance participation, we offered flexible interview timing and format and included non-English-speaking participants. Future multicenter studies are needed to overcome these limitations and strengthen the evidence base in this field.

## Conclusions

Transition from pediatric to adult HMV care is complex and challenging for patients, caregivers, and HSCPs. In this study, the need for substantial improvements in current processes were highlighted by each of these participant groups, with similar challenges and change priorities identified by those receiving and delivering medical care. Development of clear processes and standardized guidelines should be prioritized, and effort should be directed toward integrating transition processes into routine follow-up of pediatric patients from early adolescence onward. To ensure that improvements are embedded in practice and are sustainable, support at a systemic level is required.

Future studies in this field should qualitatively and quantitatively measure outcomes of transition of young adult HMV users following the implementation of formal transition frameworks and dissemination of well-designed patient- and provider-facing resources.

## Supplementary Material

aaoag108_Supplementary_Data

## References

[1] Blum RW. Introduction. Improving transition for adolescents with special health care needs from pediatric to adult-centered health care. Pediatrics. 2002;110:1301-1303. 12456948

[2] American Academy of Pediatrics; American Academy of Family Physicians; American College of Physicians–American Society of Internal Medicine. A consensus statement on health care transitions for young adults with special health care needs. Pediatrics. 2002;110:1304-1306. 10.1542/peds.110.S3.1304 12456949

[3] Amin R , MacLusky I, Zielinski D, et al Pediatric home mechanical ventilation: a Canadian Thoracic Society clinical practice guideline executive summary. Canad J Respir Crit Care Sleep Med. 2017;1:7-36. 10.1080/24745332.2017.1300463

[4] Willis LD. Transition from pediatric to adult care for young adults with chronic respiratory disease. Respir Care. 2020;65:1916-1922. 10.4187/respcare.08260 32873753

[5] MacLusky I , Keilty K. Section 12: transition from pediatric to adult care. Canad J Respir Crit Care Sleep Med. 2018;2:83-87. 10.1080/24745332.2018.1494992

[6] Dale CM , Carbone S, Amin R, et al A transition program to adult health services for teenagers receiving long-term home mechanical ventilation: a longitudinal qualitative study. Pediatr Pulmonol. 2020;55:771-779. 10.1002/ppul.24657 31971666

[7] Nakhla M , Bell LE, Wafa S, Dasgupta K. Improving the transition from pediatric to adult diabetes care: the pediatric care provider’s perspective in Quebec, Canada. BMJ Open Diabetes Res Care. 2017;5:e000390. 10.1136/bmjdrc-2017-000390 PMC5530239 28761657

[8] Jarvis SW , Roberts D, Flemming K, Richardson G, Fraser LK. Transition of children with life-limiting conditions to adult care and healthcare use: a systematic review. Pediatr Res. 2021;90:1120-1131. 10.1038/s41390-021-01396-8 PMC8671088 33654285

[9] Lindsay S. Spaces of well-being among young adults with physical disabilities transitioning from pediatric to adult healthcare. Disabil Health J. 2018;11:149-154. 10.1016/j.dhjo.2017.03.018 28592389

[10] Gurvitz MZ , Inkelas M, Lee M, Stout K, Escarce J, Chang R-K. Changes in hospitalization patterns among patients with congenital heart disease during the transition from adolescence to adulthood. J Am Coll Cardiol. 2007;49:875-882. 10.1016/j.jacc.2006.09.051 17320746

[11] Thorne S. Interpretive Description: Qualitative Research for Applied Practice. 2nd ed. Routledge; 2016:11.

[12] Srivastava SA , Elkin SL, Bilton D. The transition of adolescents with chronic respiratory illness to adult care. Paediatr Respir Rev. 2012;13:230-235; quiz 235. 10.1016/j.prrv.2012.05.004 23069122

[13] Xiao L , Amin R, Nonoyama ML. Long-term mechanical ventilation and transitions in care: a narrative review. Chron Respir Dis. 2023;20:14799731231176301. 10.1177/14799731231176301 PMC10184211 37170874

[14] Agarwal A , Willis D, Tang X, et al Transition of respiratory technology dependent patients from pediatric to adult pulmonology care. Pediatr Pulmonol. 2015;50:1294-1300. 10.1002/ppul.23155 25652000

[15] Cheng PC , Panitch HB, Hansen-Flaschen J. Transition of patients with neuromuscular disease and chronic ventilator-dependent respiratory failure from pediatric to adult pulmonary care. Paediatr Respir Rev. 2020;33:3-8. 10.1016/j.prrv.2019.03.005 31053356

[16] Dale CM , King J, Amin R, et al Health transition experiences of Canadian ventilator-assisted adolescents and their family caregivers: a qualitative interview study. Paediatr Child Health. 2017;22:277-281. 10.1093/pch/pxx079 PMC5804878 29479234

[17] Onofri A , Broomfield A, Tan HL. Transition to adult care in children on long-term ventilation. Front Pediatr. 2020;8:548839. 10.3389/fped.2020.548839 PMC7555615 33102405

[18] Toulany A , Willem Gorter J, Harrison M. A call for action: recommendations to improve transition to adult care for youth with complex health care needs. Paediatr Child Health. 2022;27:297-309. 10.1093/pch/pxac047 PMC9394635 36016593

[19] Gravelle AM , Paone M, Davidson AGF, Chilvers MA. Evaluation of a multidimensional cystic fibrosis transition program: a quality improvement initiative. J Pediatr Nurs. 2015;30:236-243. 10.1016/j.pedn.2014.06.011 25089835

[20] Domecq JP , Prutsky G, Elraiyah T, et al Patient engagement in research: a systematic review. BMC Health Serv Res. 2014;14:89. 10.1186/1472-6963-14-89 PMC3938901 24568690

[21] Leese J , Macdonald G, Kerr S, et al “Adding another spinning plate to an already busy life.” Benefits and risks in patient partner–researcher relationships: a qualitative study of patient partners’ experiences in a Canadian health research setting. BMJ Open. 2018;8:e022154. 10.1136/bmjopen-2018-022154 PMC6104752 30121606

[22] Amirnovin R , Aghamohammadi S, Riley C, Woo MS, Del Castillo S. Analysis of a pediatric home mechanical ventilator population. Respir Care. 2018;63:558-564. 10.4187/respcare.05644 29511037

[23] Pavone M , Verrillo E, Onofri A, et al Characteristics and outcomes in children on long-term mechanical ventilation: the experience of a pediatric tertiary center in Rome. Ital J Pediatr. 2020;46:12. 10.1186/s13052-020-0778-8 PMC6995086 32005269

[24] Płaszewska-Żywko L , Fajfer-Gryz I, Cichoń J, Kózka M. Burden, social support, and coping strategies in family caregivers of individuals receiving home mechanical ventilation: a cross-sectional study. BMC Nurs. 2024;23:346. 10.1186/s12912-024-02024-6 PMC11110424 38778402

